# Supplementary material for: 7T MP2RAGE for cortical myelin segmentation: Impact of aging
Source: PLoS One. 2024 Apr 16;19(4):e0299670. doi: 10.1371/journal.pone.0299670 (PMC11020839; doi:10.1371/journal.pone.0299670)
Supplement: Attachment — Submitted filename: Comments _Reviewers_Rebuttal.docx [file pone.0299670.s002.docx]

**Rebuttal**

The author would like to thank the reviewers for their insightful comments. The answers to the comments are written in Arial underneath each comment.

Reviewers' comments:

Reviewer #1: The goal of this study was to distinguish densely and sparsely myelinated sections along the cortical ribbon using novel MP2rage-derived contrasts acquired at 7T. To achieve this, the author applied k-means clustering to various combination of images and through this pure data-driven approach, they showed that contrast between high and low myelin areas could be enhanced, and the resulting segmentations were visually comparable to histology. To further explore its application potential, the author showed that separating densely vs. sparsely myelinated cortical portions provided unique information characterizing cortical changes in aging.

This study has a number of strengths in terms of imaging technique and statistical analysis, and the manuscript itself was concise and very well written. Nonetheless, I believe the paper could benefit from further refinement and there are a few areas in which the methodology is unclear as described. These concerns are detailed below.

1. The aim of this study is to separate the cortex into high vs. low myelinated “layers”. In the context of k-means clustering, the simplest implementation is to find two clusters in all cortical grey matter voxels. I’m therefore curious why the author chose to start with whole brain maps and set out to find 6 clusters instead. As a result, the current approach required several clean-up steps before arriving at the final cortical segmentations (described in 2.3.2-2.3.3). Please describe the rationale behind the current k-means approach.

Answer: Only the re-scaling step used the whole brain. The segmentation step used a SPM generated smoothed gray matter tissue map thresholded at 0.15 (region of interest mask Cortical segmentation: Raw maps) that excluded deep white matter but included superficial white matter. There were two reasons for choosing a region of interest map that also included superficial white matter and sulcal CSF. 1. Tailoring this mask to the cortical gray matter rim by choosing an arbitrary (avoiding gray/white and csf/gray partial volume voxels) threshold would mean that the segmentation of the cortical rim boundaries is largely determined by the performance of the SPM segmentation algorithm and the arbitrary threshold used for its binarization. 2. Although this project only used the cortical segmentations, the other tissue type maps and in particular the gray/white partial volume tissue map are also of interest for disease states associated with a blurring of the gray/white boundary, e.g., subtle cortical dysplasias causing epilepsy. The gray/white partial volume maps were also assessed in this project but as anticipated showed not group differences and therefore the results were not reported. Furthermore, confining the segmentation to the cortical rim would not have eliminated the clean-up steps. One of them was done to eliminate subcortical gray matter structures, e.g. thalamus, hippocampus, i.e., would in this form or another also been necessary if the segmentation would have been limited to the cortical rim. The second clean-up step was necessary to correct misclassified cortical rim voxels in regions that were affected by B1 inhomogeneities due to their closeness to the air-filled spaces, i.e., inferior temporal and orbito-frontal regions. The best way to eliminate or reduce this misclassification is to use dielectric pads in the neck and cheek regions during the image acquisition or to acquire a B1 map for correction in post-processing (Marques and Gruetter, 2013, PMID: **23874936**). The atag data does not contain a B1 map and the paper describing it (Ref 23) does not mention the use of dielectric pads. Please see also the answer to reviewer 2’s criticism No 6. The relevant parts of the manuscript were changed as follows (bold) to address the issue raised by the reviewer…

A region of interest map was generated by smoothing the UNI derived gray matter map with a Gaussian smoothing kernel of 2 by 2 by 2 mm FWHM and then binarizing it by thresholding at 0.15. **This threshold resulted in a mask that not only contained the gray matter rim but also sulcal/gyral csf and subcortical white matter as well as csf/gray, gray/white transition or partial volume regions. The reasons for choosing this larger mask instead of just a mask focusing on the cortical rim were 1. The cortical rim width is determined by k-means clustering, i.e., does not depend on the performance of the SPM segmentation algorithm. 2. Although not of interest for this project, the gray/white partial volume map may contain important information for diseases characterized by gray/white blurring, e.g., subtle cortical malformations.** This mask was used to extract the tissue intensities from each of the subject’s rescaled images, that were converted into image specific z‐scores and then alone (combinations 1 and 2) or combined with the other contrasts (combinations 3-7) supplied to the k‐means clustering algorithm implemented in MATLAB 9.4 (The Math Works, Natick, MA; number of clusters *n* = 6, squared Euclidian distance function, maximum number of iterations = 1,000, replicates = 100).

2. The author mentioned that segmentations generated from INVC and RATIO image only was noisier compared to multi-contrast combinations. When comparing different multi-contrast combinations, it was further shown that the combination of UNI, T1map, and RATIO images (all but INVC) provided better clustering results. I would therefore conclude that INVC, despite appearing to have high contrast inside cortical ribbon, is not very informative at separating myelinated areas. However, INVC was the only image used in the aging part of the study. Please justify this choice and describe in particular why RATIO is not used over INVC.

Answer: A more conservative conclusion re usefulness of INVC is that it does not improve the segmentation performance substantially beyond what can already be gained from the combination of UNI, T1map and RATIO image. Furthermore, the INVC was not the only image used in the aging part of the study. This part mostly used the low and intermediate intensity cortical rim maps derived from the combination of UNI, T1map and RATIO. The INVC and the RATIO image both allow to identify low and intermediate intensity zones visually. However in contrast to the heavily processed RATIO (skull-stropped ratio of 2 rescaled, bias corrected image), the INVC image used for the surface analysis has undergone limited processing (bias corrected ratio of two raw images) and that corresponds well to the type of processing that is necessary to create the T1/T2 ratio image. This was the main reason for choosing it over the RATIO image for the surface analysis. Furthermore, the main reason for the different performance of the INVC image in the surface-based analysis compared to the intensity maps was the limited sampling at 0.25/0.5 and 0.75 thickness. This would not have been different if the RATIO image would have been used in the aging analysis. Please see also answer to criticism 4.

3. The B1+ inhomogeneity can be much larger at 7T so the SPM bias correction within the unified segmentation routine might fail. Please include more details about this step and discuss whether voxel signal intensities may be differentially affected by this correction which in turn affecting the k-means clustering.

Answer: The MP2RAGE sequence was explicitly designed by Marques et al. (Ref 21) to reduce this particular problem. This sequence simultaneously acquires two gradient echo images with different inversion times (INV1 and INV2) but otherwise identical sequence parameters, i.e., B1^-^ , M_0_, T2* are affected in an identical matter in both images. Combining them by means of a ratio will result in an image (UNI and INVC) that is largely independent from B1^-^ , M_0_, T2*, i.e., the typical 7T bias field will be greatly reduced, i.e., is considerably less than in a 3T standard MPRAGE which was the reason why the no-ants-n4 flag had to be set for freesurfer processing. Another way to acquire bias field independent images is to perform quantitative imaging which in the case of the MP2RAGE sequence is a T1 relaxation map that can be calculated from the two INV images with the help of a look-up table (please see details in Ref 21). The issue was further investigated by the same authors (Marques and Gruetter, 2013 PLoS One . 2013 Jul 16;8(7):e69294.) who demonstrated that residual B1 inhomogeneities can be removed using the information of a B1 map. The atag data repository does not contain a whole brain B1 map and thus SPM was used to remove these residual B1 inhomogeneities. The following wording was added in “Imaging” first paragraph. to address the reviewer’s concern:

The MP2RAGE sequence generates an INV1 or TI1 gradient echo image, and an INV2 or TI2 gradient echo image. These two images can be used to calculate three additional images 1. A synthetic T1 weighted image (UNI) that is derived from the complex INV1 and INV2 data. 2. A T1 relaxation map (T1map) that can be calculated from the INV1 and INV2 images with a protocol specific look-up table (21). 3. INV1/INV2 ratio image ( INVC) that is generated by combining INV1 and INV2 using the following formula: see Figure 1).

$$INVC=\frac{INV1*INV2}{\left( INV1*INV1 \right)+\left( INV2*INV2 \right)}*100$$

Because the INV1 and INV2 are acquired under the same conditions B1^-^ , M_0_, T2* are affected in an identical matter, and therefore the UNI, T1map and INVC images derived from combining them are largely free proton density contrast, T2 contrast, reception bias field and transit field inhomogeneities.

4. The current study proposed a new way of enhancing contrast between cortical layers that can be used in aging research. More discussion comparing previous studies tackling similar problems is therefore needed for readers to evaluate the robustness and added value of current approach. Please briefly discuss current k-means segmentation results with Rowley, Front Neurosci 2015 ([https://pubmed.ncbi.nlm.nih.gov/26557052/](https://urldefense.com/v3/__https:/pubmed.ncbi.nlm.nih.gov/26557052/__;!!LQC6Cpwp!vv7y14TCWTszfG7xVLIlUnS9R3V7mSuS2nqeckDQtjKdfOFUDF1qFtM7yNG4uqFhdNu5MucKXMqjPDc5l6-0tA$)), which identified myelinated cortical thickness using T1w image, and current aging results with Sui, Neuroimage 2022 ([https://pubmed.ncbi.nlm.nih.gov/36368498/](https://urldefense.com/v3/__https:/pubmed.ncbi.nlm.nih.gov/36368498/__;!!LQC6Cpwp!vv7y14TCWTszfG7xVLIlUnS9R3V7mSuS2nqeckDQtjKdfOFUDF1qFtM7yNG4uqFhdNu5MucKXMqjPDf2mfViEA$)), which looked at depth-specific changes in normal aging.

Answer: The study of Sui et al. and their findings that are similar to the findings of this study are now mentioned in the Discussion because it further illustrates the challenges encountered with the T1/T2 ratio-based surface based approach. The study of Rowley et al is mentioned in the introduction but not discussed in detail in the Discussion section for the following reasons. 1. As mentioned in the introduction, several techniques for the imaging of cortical layers have been proposed and some of them, e.g., Viviani et al. also used a “hard” segmentation approach to split the cortex into a sparsely and heavily myelinated layers similarly as Rowley et al. and this study. Discussing them all re robustness is beyond the scope of this manuscript and doing it in a meaningful way would require applying all these techniques to the same population. 2. Rowley et al. used the method to investigate myelination patterns in dipolar disorder and not aging. That prevents comparing its findings with the findings of this papers. Interestingly the same group used an intensity approach, i.e., assessed T1 intensity at 0.25/0.5/0.75 thickness instead of the segmentation approach proposed in the 2015 paper, in a follow-up publication 2017 (PMID**: 28462512)** despite having acquired the same kind of high quality T1 data.

5. As a limitation, the author should mention that while iron and myelin tend to co-localize in the cortex, which allows T1 to be used as a myelin marker, they can dissociate in older age. Even for healthy aging population, the cortex may have iron accumulation, which has an opposite effect on T1 compared to demyelination.

Answer: The reviewer raises an important issue that has to be kept in mind when interpreting the findings of studies investigating age-associated myelin changes. The current limitation section focus on limitations of this particular manuscript. The age-associated dissociation mentioned by the reviewer is a feature that impacts not only this study but all studies investigating age and also disease related changes of the myelin signal. A multimodal approach, e.g., quantitative susceptibility mapping, would allow for a better insight into the mechanisms driving the changes observed in this study. The following limitation has been added.

6. Aging effects on cortical myelin and iron content are complex and layer-specific (53). Combining the MP2RAGE cortical segmentation approach with additional quantitative modalities, e.g., susceptibility weighted imaging, might provide additional information allowing for a more in-depth interpretation of the findings of this study.

Minor comments:
- When establishing k-means clustering for cortical segmentation, there seem to be several steps using “randomly selected subjects” (e.g., p.14: “best segmentation… was determined based on the results in 4 randomly selected subjects”). I think a more robust/convincing way might be using all subject or even k-fold cross validation but the author might have a specific reason for doing this. A brief mention of this and potential bias it might introduce may be helpful.

Answer: As outlined in the paper, it was only the first step that used a visual assessment of the segmentation quality in 4 subjects. The RATIO and INVC images in which the low and intermediate intensity zones are depicted and show a good correspondence with histological preparations were used as “ground truth” in this step. It was complemented by a quantitative approach (dice) to obtain an objective measure of the differences observed by the visual inspection. The purpose was to identify the image combination that identified the two cortical intensity zones best. A purely data driven approach as proposed by the reviewer would not have answered that question.

- There are four images in the study so when testing which combinations to use for k-means, there should be 15 combinations to choose from. Is there a particular reason why the current 7 combinations were tested? I think including a combination of UNI and T1map would also be informative but this is up to the author.

Answer: The reasons for not investigating the UNI/T1map combination were the same as the reasons for investigating the RATIO and INVC images but not the UNI and T1map as sole input for the cluster analysis. The RATIO and INVC images clearly depicted two cortical intensity zones while the UNI and T1map did not, i.e., it was assumed that the prominent INVC or RATIO contrasts “drive” the segmentation results while the UNI and T1map intensities provide confirmatory information that reduces the noise.

- Page 16 (section 2.4): “Finally, the unscaled bias corrected INVC image was used [to] investigate…”

Answer: corrected

- Page 21: “thinning” misspelled.

Answer: corrected.

Reviewer #2: Strengths:
- Compared to previous works, the quantitative nature could improve transferability
Weaknesses:
- Some of the methods and wording was unclear. I have provided suggestions to help clarify the manuscript.
- Further evidence should be provided to support the methods, or explanations as to why the chosen methods were used. My areas of concern are listed below:

Methods:
1. It should be noted that the UNI image is generated by using the complex data from the INV1 and INV2 image (equation 3 in their reference 21 - Marques et al 2010)

Answer: The information re calculation of the UNI image and the T1 map was added.

2. INVC = (INV1 *INV2)/(INV1*INV1) +(INV2*INV2))*100 -> equation provided is missing a ‘(‘ somewhere. I suspect it is supposed to be for the denominator? The author should consider writing this as an inline equation instead for improved readability as their manuscript largely revolves around its calculation.

Answer: The formula was corrected and all formulas have been written as inline equations.

3. Figure 1 -> the equation above for INVC does not match what is in the figure.

Answer: INV1/INV2 has been replaced by the text “Combination INV1 and INV2”.

4. It is not clear why the intensity needs to be adjusted/rescaled. From referencing ref 24, it doesn’t appear to do much asides from sliding the histogram, and possibly bring the intensities onto a common scale (which may be important for Ratio, but not necessarily). Has the author demonstrated that this significantly impacts the results of k-means clustering? This part of the manuscript is convoluted and requires external reading to understand the methods. In my opinion, this complication should only be included if it is essential for the results.

Answer: The re-scaling section(2.3.1) has been re-written and an additional Figure and Table have been added to explain how the re-scaling works.

5. “The signal intensities of the UNI and T1map image covary with the myelin content but while the UNI signal increases with higher myelinization, the T1 map signal decreases which results in an enhanced contrast between heavily and sparsely myelinated regions in the RATIO image (please see Figure 1).” – the use of language regarding myelination is too strong. The author already acknowledged that iron can also influence T1, so this should be reworded (see also: Stüber C, Morawski M, Schäfer A, Labadie C, Wähnert M, Leuze C, Streicher M, Barapatre N, Reimann K, Geyer S, Spemann D, Turner R. Myelin and iron concentration in the human brain: a quantitative study of MRI contrast. Neuroimage. 2014 Jun;93 Pt 1:95-106. doi: 10.1016/j.neuroimage.2014.02.026. Epub 2014 Mar 6. PMID: 24607447.)

Answer: Please see also Answer to criticism 5. The section has been extensively re-written and the statement has been deleted.

6. Can the authors further support the use of 6 clusters by reporting the summed distance to the centroid for varying numbers of clusters? This part is also unclear, what is fed into the k-means clustering? My interpretation of the reading was that the GM was masked with threshold of 0.15, and the resulting masked data is input to k-means. This should primarily leave GM and some partial volume voxels? The description for the use of 6 clusters included clusters for CSF and WM. This doesn’t appear to make sense, and the author should give further explanation and/or proof that 6 clusters is the appropriate number. It appears possible that 4 could be better (remove CSF and WM cluster), but the manuscript stated only 6 to 8 clusters were investigated.

Answer: The GM mask was smoothed map with a Gaussian smoothing kernel of 2 by 2 by 2 mm FWHM before thresholding it a 0.15. As a consequence the mask not only contained cortical rim voxels and partially volumed csf/gray and gray/white voxels but also pure subcortical white matter and sulcal csf. There were two reasons for creating this larger mask. 1. Tailoring this mask to the cortical gray matter rim would mean that the segmentation of cortical rim is largely determined by the performance of the SPM segmentation algorithm and the arbitrary threshold chosen for its binarization instead of the k-means clustering. 2. This project only used the cortical segmentations. However, the other tissue type maps and in particular the gray/white partial volume tissue map are also of interest for disease states associated with a blurring of the gray/white boundary, e.g., subtle cortical dysplasia causing epilepsy. The gray/white partial volume maps were also assessed in this project but as anticipated showed not group differences and therefore the results were not reported. The pure white and CSF maps finally allowed for a better segmentation of the partial volume maps. Taken together, the choice of 6 clusters was based on biological considerations rather than cluster centroid distances. The reason for trying out 7 and 8 clusters to find out if this would identify reliably an intermediate/low intensity partial volume zone which was not the case. The reason for trying out 3-5 clusters was to investigate how well the approach identifies the three main tissue types (3 clusters) while cluster 4 and 5 were mostly tested to learn more about how the approach identifies partial volumed clusters.

7. The subsequent paragraph in the manuscript might suggest that all the rescaling was necessary to set consistent starting guesses for centroid values. Can the author comment on this? How much are the results influenced by the input values? If they are highly influenced, it might also provide support that too many clusters were used.

Answer: Please also see answer to criticism 4. As mentioned in “Cortical Segmentation: Raw Maps”. the intensities of each image type were standardized by converting them to z-scores which also ensured that the intensities from each images were weighted equally.

8. “Effects of aging on low and intermediate intensity cortical layers were investigated with surface and volume-based analyses thus allowing for a comparison of the thinning/volume loss patterns and T peaks of the two approaches.” -> low and intermediate intensity is arbitrary as you have stated that UNI and T1 maps have opposing contrasts. The specifics under investigation should be made clearer throughout the manuscript. The author already defined inner cortical and outer cortical layers and should maintain that convention throughout. Also, it is unclear what “T peaks” is from this excerpt. It probably refers to a t-score map from a t-test comparison between young and old, but this should be explicitly stated.

Answer: The wording intermediate and low intensity zone was based on the appearance of the two zones in the INVC and RATIO images (please see Figure 1) as pointed out in “Introduction” and repeated in the first paragraph of “Conclusion. The text has been edited so that the terms low and intermediate intensity zones are consistently used when referring to the MR images.

The cited sentence was modified as follows for clarification:

Effects of aging on low and intermediate intensity cortical layers were investigated **using t-tests (young adults > older adults) with** surface- and volume-based analyses to allow for a comparison of the thinning/volume loss patterns and T peaks of the two approaches.

9. “Finally, the unscaled bias corrected INVC image was used investigate age-related myelin signal changes” – used to investigate?

Answer: The sentence has been corrected.

9. Perhaps a figure as a flowchart is necessary if all steps are deemed essential to the analysis.

Answer: It is not entirely clear if the reviewer is asking for a flow chart showing the image processing/segmentation steps or a flow chart summarizing the analysis of the resulting maps in SPM (voxel-based) and Freesurfer (surface-based). A new figure showing the processing steps and the use of the outputs for the analyses but without the processing steps in the analyses was added.

10. Can the author speak to why they chose to do two age groups (which are quite wide), compared to using a linear model with linear and quadratic age terms? Their other analysis references commonalities with surface-based approaches, so I am unclear why this has also not been maintained.

Answer: The reason for this has been mentioned in the limitation section:

The age distribution in the ATAG data set with a preponderance of subjects between 20 - 30 and 50 years and older but only few middle-aged participants did not allow for more sophisticated modelling of potential age effects.

In detail, there were 27 patients aged 19-28, mean(SD): 23.8 (2.4) years and 18 older adults aged 42-73, mean(SD) 60.5 9.8 years. There were only two subjects (42 and 45 years) in the 4^th^ and 5^th^ decade

Results:

11. I am not sure if it was a feature of the upload/transfer of files, but the brain images and segmentations in Figure 2 are blurring. Since the segmentations are binary, it would help if they were provided at a higher resolution. In its current state, it is hard to validate the results reported in the figure caption. I did download the figure, but the MRI images and segmentation are blurry.

Answer: The image formatting required by PLOS ONE required a down-sampling of all figures. An improved version of the Figure has been uploaded.

12. Figure 4 – I cannot make sense of the figure. The colour bar goes hot and cold, which should remove the need for the two separate directional comparisons. I.E. everything in the bottom plots should be in the top plots using the cold end of the colormap. Or remove the cold side of the colour map. Or a clearer description needs to be made of what is being presented.

Answer: The color bar has been adapted to show only thinning in red.

Discussion

13. The author keeps referring to cytoarchitecture, but seemingly provided no evidence to support the correlation between that and MR. The MR contrasts seem to primarily follow myeloarchitecture patterns.

Answer: Please see Figure 3 for evidence how myeloarchitecture and cytoarchitecture relate to each other and why the first might allow for assumptions re composition of the latter.

14. It is known that MP2RAGE is impacted by B1 inhomogeneity. The authors should discuss the potential impact of this on their findings. Marques, Jose P., and Rolf Gruetter. "New developments and applications of the MP2RAGE sequence-focusing the contrast and high spatial resolution R1 mapping." PloS one 8.7 (2013): e69294. Haast, Roy AM, et al. "Effects of MP2RAGE B1+ sensitivity on inter-site T1 reproducibility and hippocampal morphometry at 7T." Neuroimage 224 (2021): 117373.
Of particular note, the T map clusters in Figure 3 for the surface analysis appear similar to B1 inhomogeneity maps. Lower intensity layers were significant where B1+ is high, and intermediate layers were significant were B1+ is classically low. Could this be related to the ambiguity in classification of layers 3+ 4 mentioned in discussion?

Answer: As stated by Marques et al. the best way to reduce B1 inhomogeneities is to acquire a B1 map or to use dielectric pads. The atag project did not acquire B1 maps and the paper describing the repository does not mention the use of dielectric pads. Regions where B1 was too low to meet adiabatic conditions affected the mid-temporal inferior temporal gyrus (similarly as shown in Fig 7 of Marques et al.) and were more prominent on the right side. As mentioned in the manuscript (Cortical Segmentation: Final maps), brain regions above the os petrosum and sinus sphenoidalis are also affected by B1 inhomogeneities which impact the segmentation performance and required an additional clean-up step. This has been mentioned in the manuscript in the discussion as limitation (No 3) and acknowledges that this might prevent the detection of findings in these regions. The localization of these effects depends on the coil and as shown by Marques et al. on the head positioning and head size/shape. According to the atag paper, the same coil was used for all participants. Head positioning is usually standardized in research institutions and the images suggest that this was also the case for the images acquired for the atag data repository. It seems unlikely that the low intensity findings in Figure 3 are driven by B1 inhomogeneities because a. in contrast to the inferior temporal gyrus, the posterior parahippocampal gyrus was not affected by these inhomogeneities. b. It can be assumed that the B1 inhomogeneity pattern is similar in both groups because it seems unlikely that one group had a systematically larger head or different head shape that would have resulted in a significant group difference in the B1 inhomogenity fields.

15. The authors used a “b1 correction” algorithm that aims to sharpen the histogram of each tissue type. Does the author believe that this is a necessary step instead of using a B1 map-based correction? It is likely this was done as such due to the data availability, but it is worth a discussion point.

Answer: It is assumed the reviewer refers to the use of the SPM bias correction algorithm that removes slowly varying intensity inhomogeneities. It mostly affected intensities of “deep” brain structures, e.g. deep white matter or deep gray matter structures, e.g., thalamus. Since the gray matter mask included deep gray matter structures using SPM for additional bias correction was deemed beneficial but was not formally tested.

16. The work presented in this manuscript appears to be an extension of work previously done using only a T1-weighted contrast. The authors should discuss how their work compares and advances findings over this methodology: Rowley, C. D., Bazin, P. L., Tardif, C. L., Sehmbi, M., Hashim, E., Zaharieva, N., ... & Bock, N. A. (2015). Assessing intracortical myelin in the living human brain using myelinated cortical thickness. Frontiers in neuroscience, 9, 396.

Answer: Please see also answer to reviewer 1’s criticism 4. The paper has now been added to the reference list together with several other approaches to segment the cortical rim. Although there are commonalities, e.g., “hard” or binary segmentation of heavily and sparsely myelinated rim zones, there are also many differences, e.g., combination of several 3T BRAVO sequences with 1 mm resolution, fuzzy c-clustering with probabilistic segmentation that required arbitrary thresholding, 3 clusters (white matter, myelinated GM and unmyelinated GM) without partial volume and or CSF clusters which means that the CSF/GM boundary was determined by the MDGM segmentation algorithm and not by the fuzzy c-clustering, application in dipolar disorder etc. While it is possible to list all these differences it is difficult to meaningfully comment on the weaknesses and strengths of two quite different approaches and would raise the question why this paper was chosen and not others, e.g. Viviani et al.. A comprehensive review of all the approaches mentioned in the Introduction is better suited for a review paper on this topic though.

Minor Comments:
MP2RAGE is classically written using all capital letters. I suggest that this convention is maintained in the current manuscript.

This has been corrected
