# Supplementary material for: 7T MP2RAGE for cortical myelin segmentation: Impact of aging
Source: PLoS One. 2024 Apr 16;19(4):e0299670. doi: 10.1371/journal.pone.0299670 (PMC11020839; doi:10.1371/journal.pone.0299670)
Supplement: Attachment — Submitted filename: Comments_reviewers_rebuttal2.docx [file pone.0299670.s003.docx]

**Rebuttal**

Again, the author would like to thank the reviewers for their insightful comments.

Reviewer #1: 

Right before “Image Processing”: …therefore the three images derived from combining them are largely free [of] proton density contrast…

**Answer**: The typo has been corrected.

Reviewer #2:

I appreciate the time the author took to respond to my initial concerns and have addressed a few of them in this revision. The inline equations and flow chart have helped to clarify the methods. However, I found some of the responses to not fully address other points. I have listed them below:

1. At a high-level, the present manuscript is suggesting to segment the cortical grey matter into superficial and deep layers using a k-means clustering approach. This intended outcome is equivalent to Rowley et al 2015, but the approach to get there is different, in part due to having different data.
The author has stated in this revision as to some reasons that they may feel that their new method is superior to the previous method, and it may very well be. It appears the dilation of the GM mask and subsequent re-segmentation with extra clusters could add robustness to the current method. Add to this that the method is intending to use a quantitative metric that should remove additional variance in the image, hopefully increasing its transferability. All these things however appear to be small methodological improvements on a previously reported method with the same application. This comes to light as the last sentence in the first paragraph of the discussion presents this as a completely new method: “The next paragraphs will discuss the new method and the findings in more detail.”

**Answer:** As pointed out by the reviewer, there was no formal comparison between the method proposed here and those proposed by Rowley et al. 2015 and Viviani et al 2017. So it is assumed that the reviewer refers to the comments in the rebuttal letter. This section only listed the differences between the method introduced by Rowley and in no way mentioned that one method is better than the other. In contrary, it explicitly stated that a meaningful comparison, i.e., a comparison that would include an evidence based judgement of superior performance, is not possible without either at least being able to work with the same study population and/or having the ground truth information, i.e., histological confirmation, for each data set. This is not possible with the available data. Finally, the last remark re “new method” depends on a person’s definition of new because there exist no objective benchmarks for what has to be deemed a new or just an alternative method. Now that short summaries of the methods proposed by Rowley et al. and Viviani et. al. have been added, the readers can decide for themselves if the method proposed here is sufficiently different to be considered new or just an alternative.

2. The general comment is that the author includes a lot of introduction and discussion on points that the MRI resolution is unable to resolve, such as the in-depth discussion on the components of different cortical layers. This space would be better utilized discussing how this method relates and improves upon previous ones. Ultimately, this is a manuscript that is proposing a new method, with the aging-information just providing evidence of its utility. At minimum, an additional paragraph in the discussion is warranted to compare this current method to others that aim to do this exact thing. This should also include Viviani et al 2017, as the author mentions, which instead uses Gaussian mixture modeling, but adds in additional contrasts to improve the segmentation. The author needs to provide a critical comparison of their method to the others, otherwise, why did they not use what was previously developed? This question needs to be addressed in the manuscript.

**Answer:** A short discussion of the methods proposed by Rowley et al and Viviani et al has been added to the discussion. A stated before, it is not possible to provide a meaningful comparison of the three methods beyond listing the technical differences. As to the last question, why not use a previously developed method? This is an interesting question particularly in a field that has at least 6 different T1 based methods to measure cortical thickness (FreeSurfer, CIVET, Brainvoyager, Brainsuite, CAT, ANTS) that are all used by the community.

3. Abstract:
“Myelin and iron are major contributor to” – plural

**Answer:** The typo has been corrected.

4. Introduction:
“Cortical myelin and iron, that often co-localizes with cortical myelin, represent the main components of the cortical MR signal (11-13)” – rewrite

**Answer:** The statement has been re-written

5. “The second aim was to compare the resulting segmentations with histological myelin maps from the literature (4) to confirm that the two zones indeed represent heavily and sparsely myelinated” – given the data is from separate individuals, you are unable to ‘confirm’ this. You are providing support that it is likely that. This is mentioned in the limitations in the discussion/conclusion but should also me clarified here.

**Answer:** The sentence has been re-written.

6. Methods:
Figure 2 caption – “e. Raw segmentations produced by c-means clustering. f. final segmentations after clean-up” - k-means?

**Answer:** The typo has been corrected

7. In the pre-processing section, the author should cite the paper for the unified segmentation method - J. Ashburner and K.J. Friston. Unified segmentation. NeuroImage, 26:839–851, 2005.

**Answer:** The reference has been added. It should be pointed out though the “segment” as it is currently implemented in SPM12 has been modified quite a bit since the 2005 publication.

8. I still believe that the scaling is adding unnecessary steps to this method. The crucial point that needs to be addressed appears to be: “is the absolute value of the images important for the processing”.
The current methodology is simply applying a linear scale factor, globally across the image. Ref1 and Ref2 are each a single number that was empirically determined. The CSF and WM mode values are also single values. So, the resulting scaling factor is a single value that is applied across the whole image. The histograms in Figure 3A and Figure3B are identical if you ignore the numbers on the x-axis. The images that are displayed in Figure 3A and B would also look equivalent if the window and level values set for the 3A portion were scaled by the values used to rescale the image. So overall, this adds an additional step that changes nothing but the values globally in the image. The peaks pointed out do move, but this should give you a RATIO histogram with the same distribution that is scaled by (UNIfact/T1fact). This is why the contrast is unchanged in the image, but the range is ‘flattened’, as the author states. In the first review, I asked for the author to provide a reason for the rescaling of images, and it is possibly clearer now that it is unnecessary. Which means that this statement would not true:
“This had a major impact on the contrast behavior of the RATIO image calculated from these two images. Compared to the RATIO image calculated from the non-rescaled images whose contrast behavior was similar to the UNI image, the image calculated from the re-scaled had an enhanced and “inverted” contrast between heavily and sparsely myelinated regions”
If I am incorrect, then the author should add the histogram for the RATIO image to Figure 3 so that the reader can see this, so they don’t have the same confusion as I have. Referencing their previous work (ref 25), they also displayed histograms for rescaled UNI and T1 maps. Interestingly, in [25] there was also a shift value which appears to have been dropped in this work. The shift is also a global shift, so even it was applied, the scaled ratio would have the same shape with global change in values of ([UNIfact+UNIshift] / [T1fact+T1shift]). The increase in dynamic range could be useful for presetting values for visualization software, or for limiting rounding problems, if the data is strictly forced to be integer values. However, given the histogram shape doesn’t change, and the relative difference between tissue types also goes unchanged, it should not impact the results of the k-means clustering if the data lives in Matlab in double format (standard for matlab processing). I think this is important to work out, as the author is claiming that this is one of their novel contributions to the advancement of being able to categorize the cortex into two layers with MRI.

**Answer:** The reviewer correctly states that the scaling factor is a single value for each image. In the case of the images displayed in Fig 3, the UNI scale factor is 0.0248 and the T1 map scale factor is 0.0288. Multiplying the image intensities with these scale factors reduces the dynamic range of both images while maintaining the overall shape of the histograms but it should not introduce a shift and it also does not explain why the gray/white ratios in Table 1 are slightly different. This is further supported by the fact that neither the RATIO histograms nor the images (please see enlarged cortical rim sections) generated from the non-scaled and scaled images look the same. Taken together, these differences suggest that the re-scaling does not affect all intensities in the way the reviewer suggested. To illustrate this point the raw segmentations generated from the images depicted in Figure 3 were used to extract the intensities of the 5 tissue maps, i.e., csf/gm partial volume (csf/gm), intermediate intensity gray matter zone (int), low intensity gray matter zone (low), gm/wm partial volume (gm/wm) and subcortical white matter (wm). The histograms of these tissue map intensities were combined with the histogram of the whole image histogram. If the re-scaling affects all tissue intensities in the same way one would expect that their histograms look similar in the non-scaled and scaled image with the range being the only difference. However, this is not the case. The most striking differences are seen for the int histograms. The int histogram of the scaled T1 map image has a higher peak with a steeper decline towards higher intensities than the histogram of the non-scaled T1 map indicating a higher number of int voxels in the lower intensity range in the former. The int histogram of the scaled UNI image also has a higher peak compared to the non-scaled UNI but here the scaling increased the number of voxels in the higher intensity range. As a result, the int voxels of the scaled RATIO image are visually brighter than those in the non-scaled image. The scaling effect on the low histogram is very subtle and thus its intensity behavior in the scaled and non-scaled images similar. The different scaling behavior of the int and low intensity voxels is best illustrated by the fact that they switch positions in the scaled RATIO histogram, i.e., the int histogram is on the left side of the low intensity histogram in the non-scaled RATIO image but on the right side in the scaled RATIO image. Based on this the conclusion that the scaling is necessary and that this is most obvious in the RATIO image seems justified. The main effect of the re-scaling is to improve the segmentation of the low intensity cortical zone.

The reviewer is correct that [25] also calculates an image specific left shift and that this is not done in this project. Table 1 in [25] shows that the combined effect is an increased gray/white contrast which improves the segmentation of internal brainstem structures that have lower gray/white contrasts than cortical gray/subcortical white matter that is already strong in the UNI and T1 maps.

9. It is also worth noting that the UNI image by definition should range from -0.5 to 0.5 (see Marques et al 2010), which was partially done to have a standardized way to set color bar limits for visualization (see my point above). It appears the author is using a version from the scanner that is scaled from 0-4096. But this is an important point to bring up here, as the argument I am making is that the absolute values of the metrics moving forwards from this step should not matter, and thus the rescaling is unnecessary. If the absolute value of the resulting image is necessary for the subsequent steps, then this range should be fixed (i.e. from -0.5 to 0.5).

**Answer**: The reviewer’s observation is correct. The UNI image calculated by the wip and product version of the MP2RAGE sequence have a larger dynamic range than the images used by Marques et al. 2010. Please see answer to criticism 8 why the re-scaling is not superfluous.

10. It also appears that the next step of the processing is to z-score these values, which further suggests that this global scaling measure in not needed.

**Answer:** The three input images (re-scaled UNI, T1 map and RATIO) have different dynamic ranges, i.e., the range of the RATIO image is smaller than that of the T1 map and UNI image which results in unequal variances. K-means clustering puts more weight on variables with smaller variances and z-scoring is a way to prevent that, i.e., z-scoring is used to standardize the data.

11. Discussion:
“the potential to provide layer-specific cortical volumetric information”- Needs to be reworded or be made clearer, as you cannot provide layer-specific information for a 6(+) layer tissue, with only two clustered layers. Additionally, the author provides discussion on this exact point of resolution and the ambiguity of where layers 3 and 4 end up yet starts the following paragraph with: “The new layer specific approach’s potential…”.

**Answer**: The sentence refers to the “potential”, it does not state that it provides 6 layer information. Furthermore, as the reviewer states themselves, large parts of the discussion discuss the limitations of the intermediate/low intensity zone so if it is assumed that the reader does not just skim the Discussion there should be no misunderstanding that it is not possible to depict the 6 layer structure with this approach.

Minor:
Many locations the author listed “k-mean” but I believe it should be “k-means”.

**Answer**: Two instances of k-mean have been corrected to k-means.
