# Supplementary material for: 7T MP2RAGE for cortical myelin segmentation: Impact of aging
Source: PLoS One. 2024 Apr 16;19(4):e0299670. doi: 10.1371/journal.pone.0299670 (PMC11020839; doi:10.1371/journal.pone.0299670)
Supplement: Attachment — Submitted filename: Comments_reviewers_rebuttal3.docx [file pone.0299670.s004.docx]

**Rebuttal**

The author would like to thank the editor for the chance to submit a third revision and the reviewer for their insightful comments that helped to identify and amend a major issue in the previous versions.

Reviewer #2: I appreciate the time the author took to respond to my initial concerns and have addressed many of them in this revision. The redraft of Figure 3 I think is great, but it appears to highlight an error in the data processing. I cannot recommend this manuscript for publication until this is addressed.

For the problem, it is best to put numbers to this to explain.
I took approximate mean values for each tissue class (in order of WM, GM/WM, GM, low, int, csg/gm) from this histogram and calculated the results myself. The top row all looks correct (unscaled), and the approximate values I pulled are:
T1: 1250, 1500, 1800, 2200, 3000
UNI: 3200, 2700, 2000, 1200, 700
RATIO: 250, 175, 100, 50, 25

If I calculate the ratio based on UNI/T1*100 values I pulled from the histogram, I get: 256, 180, 111, 54, 23 (within rounding error of the histogram RATIO values). Everything looks in order.

If we move to the second row with the scaled values, the author reported the scaling factor was 0.0248 for UNI, and 0.0288 for T1. If I apply that to the above numbers, I get good agreement in the T1scaled and UNIscaled histograms which have approximate values of (again in order of WM, GM/WM, GM, low, int, csf/gm):
T1: 30, 45, 55, 70, 90
UNI: 80, 65, 45, 30, 10
If you take the ratio of this “UNIscale/T1scale *100”, you get:
My calculated RATIO: 260, 144, 81, 42, 11 -> these are all close to the first set, but IMPORTANTLY are monotonically decreasing. So, it should be impossible to get the characteristic dark ring at the border of the WM/GM.

The values reported for the histogram in Figure 3, which match the image displayed are
Figure 3 bottom RATIO: 40, 20, 5, 15, 20

Two obvious problems, 1. They are no longer monotonically decreasing. And 2. None of them match the very simple mathematical operation that is being reported. Perhaps with this, the author can realize my stance that the scaling operation is not needed. But it appears that another image entirely is being used to generate this contrast.

The only thing I can think of, is that this ratio is actually INV1/INV2, or something along those lines. Or you have subtracted an offset in the calculation of the ratio, and then taken the absolute value, to fold the negative values back around. Either way, the author needs to determine what has gone wrong in their processing scripts, and properly reflect their processing methods in the Methods section. This may impact other areas of the manuscript as well.

Another issue that sticks out to me is the change in tissue distributions in the histogram. The change in relative peak heights seems problematic unless variable bar widths are being used. It looks to me as though more voxels are now being counted as being in the ‘int’ tissue class and less in the “low” on the scaled UNI and T1, than in the original. The same segmentation should be used for both, such that the total voxels belonging to a tissue class are consistent. If they are not, then the claim should be more that the different intensity changes the segmentation, rather than the suggesting the intensity itself is changing.

Answer: The reviewer is correct. The unexpected behavior (no longer monotonically decreasing histograms) has been caused by a one letter typo in the Matlab code that meant that the script calculated the ratio between INVC and T1map instead of the ratio between UNI and T1map. The latter indeed behaves as predicted by the reviewer and confirms the reasoning that the scaling is not necessary. The scaling step was therefore eliminated from the processing routine. The next step was then to investigate the performance of the true UNI/T1map image and compare it to that of the accidentally calculated INVC/T1map image. This was done by first visually investigating the impact of the two ratios non-scaled ratios, i.e., INVC/T1map (RATIO) and UNI/T1map (sRATIO) on segmentation performance when used as sole input (RATIO, sRATIO, INVC) and of all combinations of these two contrasts with the remaining contrasts. Combinations that were able to identify the 6 tissue categories (csf, csf-gm partial volume, intermediate intensity gray matter rim zone, low intensity gray matter rim zone, gray matter-white matter partial volume and superficial white matter in all test subjects were further evaluated by calculating the mean within cluster (voxel to centroid of cluster it was assigned) and mean between cluster (voxel to all other cluster centroids) distances. Using this metric the contrast combination RATIO, T1map, UNI performed best, closely followed by RATIO, T1map, sRATIO. The data was reprocessed without re-scaling and all analyses repeated. As expected, the findings did not change.

The contrast combination of sRATIO, T1map and UNI was able to segment the 6 tissue categories but the map with the low intensity gray matter rim zone contained a prominent outer ring corresponding to dura residuals that was so consistent across subjects that it affected the template creation in DARTEL. Considering the inferior performance of the originally intended contrast sRATIO compared to the mistakenly calculated RATIO, the typo turned out to be serendipitous. The manuscript text was re-written to incorporate the new findings.

Minor:
Introduction:
“Cortical myelin and iron, that often co-localizes with cortical myelin, ….” – I mentioned this last review, and it still doesn’t make sense to say that cortical myelin co-localizes with itself. Perhaps you mean to say “Iron, which often co-localizes with cortical myelin,…”.

Answer: “that” in this sentence refers only to iron and not also to the myelin in the main sentence. Similarly as ….the man and the dog, that barked at all other dogs, went home… does not mean that the man and the dog are both barking at other dogs. This difference in interpretation was the reason that the author did not understand the issue raised by the reviewer. The sentence was re-written to eliminate any possibility of a misunderstanding.
